# Over-expression of EGFR regulated by RARA contributes to 5-FU resistance in colon cancer

**DOI:** 10.18632/aging.102607

**Published:** 2020-01-02

**Authors:** Xin-Yue Gu, Yang Jiang, Ming-Qi Li, Peng Han, Yan-Long Liu, Bin-Bin Cui

**Affiliations:** 1 Department of Colorectal Surgery, Harbin Medical University Cancer Hospital, Harbin 150040, People’s Republic of China; 2 Department of Pathology, Harbin Medical University Cancer Hospital, Harbin 150040, People’s Republic of China

**Keywords:** autophagy, drug sensitivity, EGFR, 5-fluorouracil

## Abstract

A promising new strategy for cancer therapy is to target the autophagic pathway. However, comprehensive characterization of autophagy genes and their clinical relevance in cancer is still lacking. Here, we systematically characterized alterations of autophagy genes in multiple cancer lines by analyzing data from The Cancer Genome Atlas and CellMiner database. Interactions between autophagy genes and clinically actionable genes (CAGs) were identified by analyzing co-expression, protein-protein interactions (PPIs) and transcription factor (TF) data. A key subnetwork was identified that included 18 autophagy genes and 22 CAGs linked by 28 PPI pairs and 1 TF-target pair, which was EGFR targeted by RARA. Alterations in the expression of autophagy genes were associated with patient survival in multiple cancer types. RARA and EGFR were associated with worse survival in colorectal cancer patients. The regulatory role of EGFR in 5-FU resistance was validated in colon cancer cells in vivo and in vitro. EGFR contributed to 5-FU resistance in colon cancer cells through autophagy induction, and EGFR overexpression in 5-FU resistant colon cancer was regulated by RARA. The present study provides a comprehensive analysis of autophagy in different cancer cell lines and highlights the potential clinical utility of targeting autophagy genes.

## INTRODUCTION

Autophagy is a highly conserved mechanism of self-digestion that removes damaged organelles and proteins from cells. However, the role of autophagy in the regulation of programmed cell death is incompletely understood. Autophagy may either directly lead to cell death (autophagic cell death) or modulate apoptosis via autophagy-apoptosis crosstalk [1, 2]. Autophagy plays an important role in maintaining cellular homeostasis and is therefore constitutively active at a basal level in most cell types [3]. Under conditions of stress, this process is used by cells to recover from homeostatic disturbance, and the system is therefore maintained in standby mode. Malignant cells undergo substantial stress in patients receiving chemotherapy, and tumor cells may rely on autophagy to eliminate the drug or resist drug cytotoxicity [4]. Although the role of autophagic cell death remains controversial in cancer, autophagy plays a cytoprotective role, promoting cell survival against apoptosis during chemotherapy treatment. Dysregulation of the autophagy pathway in cancer cells plays a role in tumor dormancy and radio- or chemoresistance [5]. Indeed, cancer was the first disease associated with alterations in autophagy as well as the first for which clinical trials in humans were performed [3].

Protein degradation occurs through the formation of autophagosomes, which are characterized by a double membrane vesicle that sequesters part of the cytoplasm. The formation of autophagosomes is initiated by the induction of various autophagy-related genes, including microtubule-associated protein 1 light chain 3, phosphatidylinositide 3 kinase (PI3K), Beclin-1, and ATG genes. ATG genes involved in cancer have been identified [6]. Autophagy plays a complex dual role in tumorigenesis, which makes the development of autophagy-based cancer treatments challenging. Yang et al. showed that increased autophagy levels in mouse pancreatic cancer lead to tumor regression and a prolonged lifespan [7]. However, ATG5 is overexpressed in gastric [8] and prostate [9] cancers, whereas ATG7 is overexpressed in bladder cancer [10]. These results demonstrate the involvement of core ATG genes in tumor development and progression, and activation or inactivation of autophagy can contribute differently to tumorigenesis according to tumor type and developmental stage.

Therefore, targeting ATG genes in cancer holds great promise. Various strategies have been investigated to explore the potential of silencing ATG genes as a putative anticancer strategy. Inhibiting autophagy using anti-malarial compounds such as chloroquine and hydroxychloroquine in combination with frontline therapeutic agents such as cisplatin and taxol results in significant inhibition of tumor growth [11]. Furthermore, genetic silencing of key ATG proteins such as Beclin 1 favors survival and decreases resistance to chemotherapy [12–14]. High Beclin 1 and LC3 levels in ovarian tumors are associated with improved overall survival [15]. Therefore, the modulation of ATG genes in response to therapeutic agents could have anti-cancer efficacy and decrease therapy resistance.

Here, we identified autophagy-related genes associated with drug sensitivity in pan-cancer and examined the association with clinically actionable genes (CAG). We focused on the ATG gene EGFR and its transcription factor (TF) RARA by analyzing of drug sensitivity-related key sub-networks. The role of EGFR and RARA in 5-fluorouracil (5-FU) resistant COAD (colorectal cancer) cells was analyzed. The present results provide a systematic analysis of ATG genes across different cancer cell lines and highlight the significant roles of autophagy in cancer therapy.

## RESULTS

### Potential effects of ATG genes on drug sensitivity

**Figure 1:**
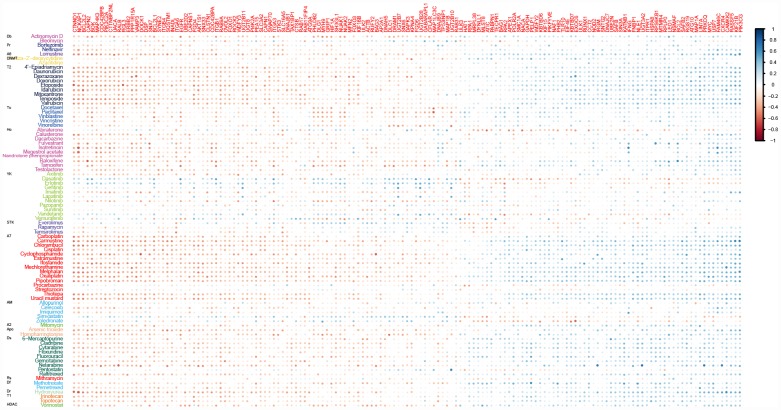
**Correlations between drug sensitivity and the expression of ATG genes for at least ten drugs.** Color bars indicate the Pearson’s correlation coefficient (PCC) between drugs and ATG genes. Different colors represent drugs with different MOA values. MOA: A2: alkylating at N-2 position of guanine; A6: alkylating at O-6 of guanine; A7: alkylating at N-7 position of guanine; AM: antimetabolite; Apo: apoptosis inducer; Db: DNA binder; Df: antifols (impairs the function of folic acids, which inhibits production of DNA, RNA, and proteins); DNMT: DNA methyltransferase inhibitor; Dr: ribonucleotide reductase inhibitor; Ds: DNA synthesis inhibitor; HDAC: Histone deacetylase; Ho: hormone; Pr: protease/proteasome; Rs: RNA synthesis inhibitor; STK: serine/threonine kinase inhibitor; T1: topoisomerase 1 inhibitor; T2: topoisomerase 2 inhibitor; Tu: tubulin-active antimitotic; YK: tyrosine kinase inhibitor.

To evaluate the potential effects of ATG genes on drug response, we tested for a correlation between the sensitivity of 84 anticancer drugs and the transcriptional expression of 770 ATG genes based on NCI60 cancer cell lines from CellMiner database using PCC (Supplementary Figure 1). We identified 2667 correlation pairs between drug sensitivity and the transcriptional expression of 151 ATG genes after filtration (Figure 1). Previous studies showed that several drugs could trigger autophagy in tumor cells, such as Melphalan, 5-FU, and docetaxel [16]. Melphalan, a DNA-damaging drug, induces caspase-dependent apoptosis and concurrently triggers Beclin 1-regulated autophagy in human Beclin 1 positive cell lines [17]. These observations were confirmed in the present study. Melphalan was associated with more than half of the ATG genes identified, including 42 negative correlations and 38 positive correlations. Sensitivity of 5-FU (negative correlation) was associated with 37 ATG genes, and resistance (positive correlation) was associated with 14 ATG genes. Treatment with 5-FU may mediate autophagy turnover both *in vitro* and *in vivo* [18]. The present results indicated that other drugs may also trigger autophagy. For example, Pipobroman, an anti-cancer drug that probably acts as an alkylating agent, was correlated with the expression of up to 70 ATG genes.

### Interactions between clinically actionable genes and ATG genes

**Figure 2:**
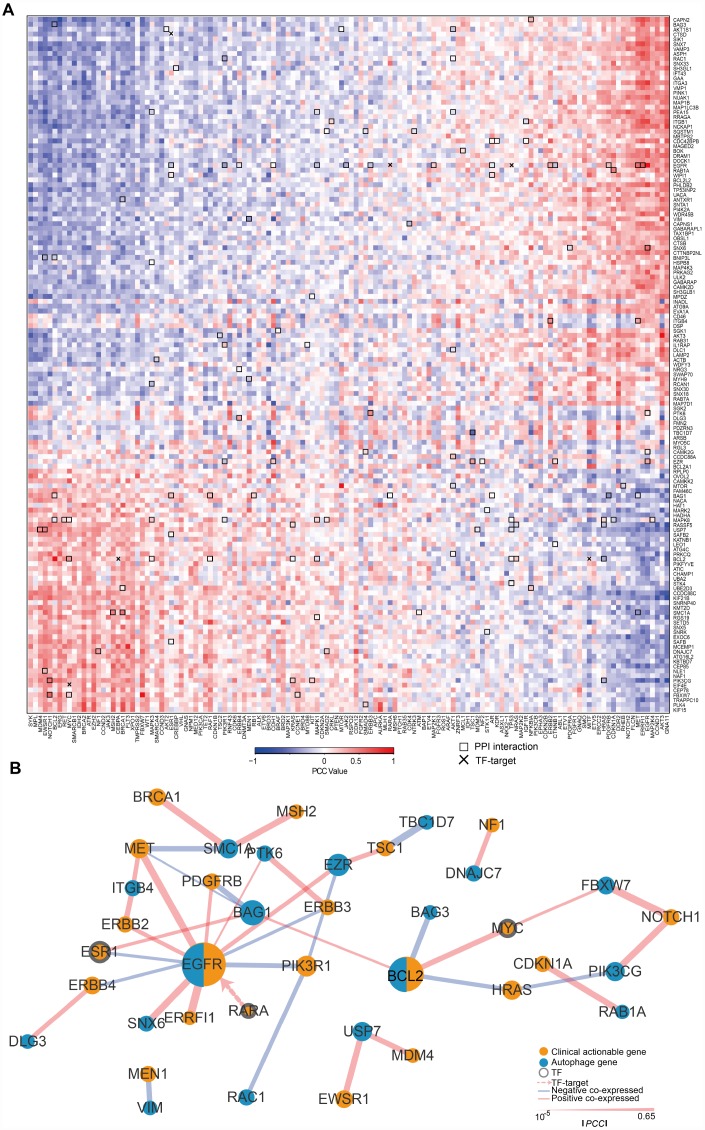
**The expression of ATG genes is associated with clinically actionable genes in cancer cell lines.** (**A**) Correlation between the expression of ATG genes and clinically actionable genes (CAGs). Blue, negative correlation; red, positive correlation. Bold boxes highlight the protein-protein interactions of actionable genes and ATG genes based on HPRD. The x marks transcription factor (TF)-target relationships for CAGs and ATG genes. Color bars indicate the PCC between drugs and autophagy genes. (**B**) Sub-network by PCC |R| > 0.3; *p* <0.05. Orange, CAGs; Blue, autophagy genes. The width of the edge represents the PCC (the bolder the higher).

To understand the clinical implications of the ATG genes, we examined the correlations between the transcriptional expression of ATG genes and 132 CAGs (targets of FDA-approved drugs or their related marker genes). First the PCC between ATG genes and CAGs (Figure 2A) and 3895 pairs with a |PCC| > 0.3 were identified. All the CAGs had significant correlations with ATGs. The number of CAGs significantly correlated with ATG genes ranged from 3 to 103 (|PCC| > 0.3, p < 0.05). The number of autophagy gene significantly correlated with CAGs ranged from 21 to 45 (|PCC| > 0.3, p < 0.05). For example, CDC42BPB showed a significantly negative correlation with 25 CAGs genes enriched in key signaling pathways, such as PI3K/AKT, p53 signaling pathway, and microRNAs in cancer pathways (Supplementary Figure 2A). KIF21B showed a significant positive correlation with 23 CAGs enriched in many cancer-related pathways such as thyroid cancer, small cell lung cancer and central carbon metabolism in cancer (Supplementary Figure 2B), suggesting that KIF21B plays a role in the development of various types of cancer.

To further investigate the interactions between ATG genes and CAGs, we detected the regulatory relationships using PPI and TF-target data (Figure 2A). A key sub-network was identified, including 28 PPI pairs and 1 TF-target pair, namely, EGFR targeted by RARA. There were 18 ATG genes and 22 CAGs (|PCC| > 0.3, p < 0.05, Figure 2B) in the sub-network. Among them, EGFR and BCL2 acted as ATG genes, and are also CAGs. EGFR was the hub node with the highest degree in the sub-network.

These results suggested that ATG genes are potentially regulated by CAGs, and highlighted the significance of autophagy in cancer treatment. Therefore, significant interactions between CAGs and ATG genes may affect drug responses and should be considered in cancer therapy.

### Clinical relevance of ATG genes

**Figure 3:**
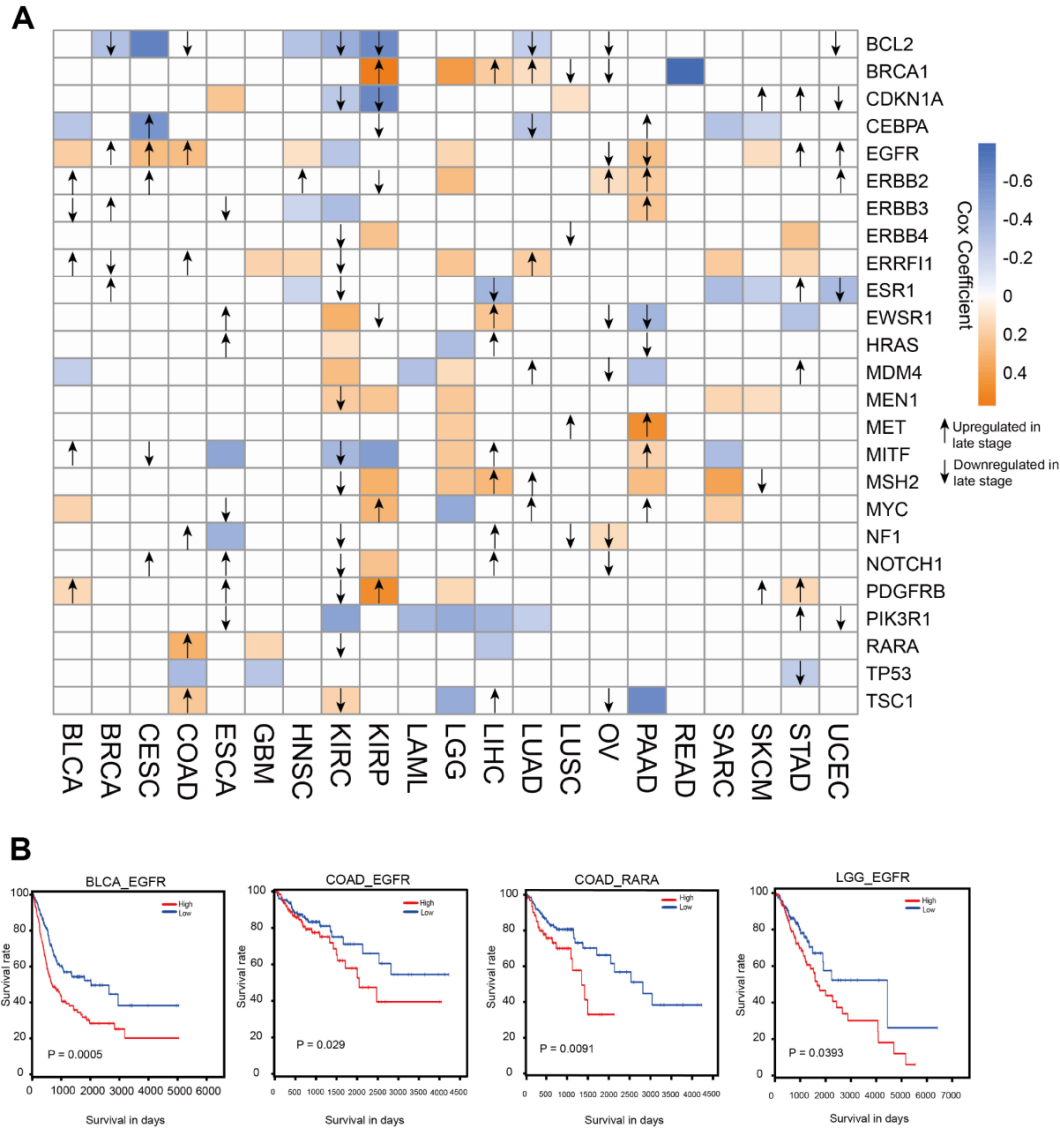
**Clinical relevance of ATG genes in different cancer types.** (**A**) Clinically relevant ATG genes in different cancer types. The red and blue boxes indicate high and low expression in tumors associated with worse overall survival times (log rank test *p* <0.05), respectively. The arrows represent the up- or downregulation of genes in later stages. (**B**) Kaplan-Meier curves of multiple cancer types stratified by median expression levels of ATG genes.

Because ATG genes often show alterations in cancer, they could provide important information for translational medicine. Here, we investigated the associations between ATG genes and overall patient survival in at least one cancer type using the 38 genes identified in the key sub-network (Figure 3A). Several ATG genes showed oncogenic features. For example, PDGFRB overexpression was significantly associated with poor survival in stomach adenocarcinoma (STAD) (log rank test p=0.016), brain lower grade glioma (LGG) (log rank test p=0.029), kidney renal papillary cell carcinoma (KIRP) (log rank test p=0.00004) and bladder urothelial carcinoma (BLCA) (log rank test p=0.0076). By contrast, several other ATG genes showed potential tumor suppressor features, such as TP53. Certain ATG genes showed different properties in different cancers. For example, EGFR over-expression was associated with poor survival in SKCM, PAAD, LGG, COAD, HNSC, CESC, and BLCA, whereas its down-regulation was associated with poor survival in KIRC. Overexpression of RARA, the only TF for EGFR in the sub-network, was associated with poor survival in COAD patients (Figure 3B). These results suggested the potential involvement of ATG genes in tumor progression and also indicated that the TF-target relationship between EGFR and RARA may play a key role in COAD. We therefore focused on EGFR in subsequent experiments.

### EGFR contributes 5-FU resistance in colon cancer cells through autophagy induction

**Figure 4:**
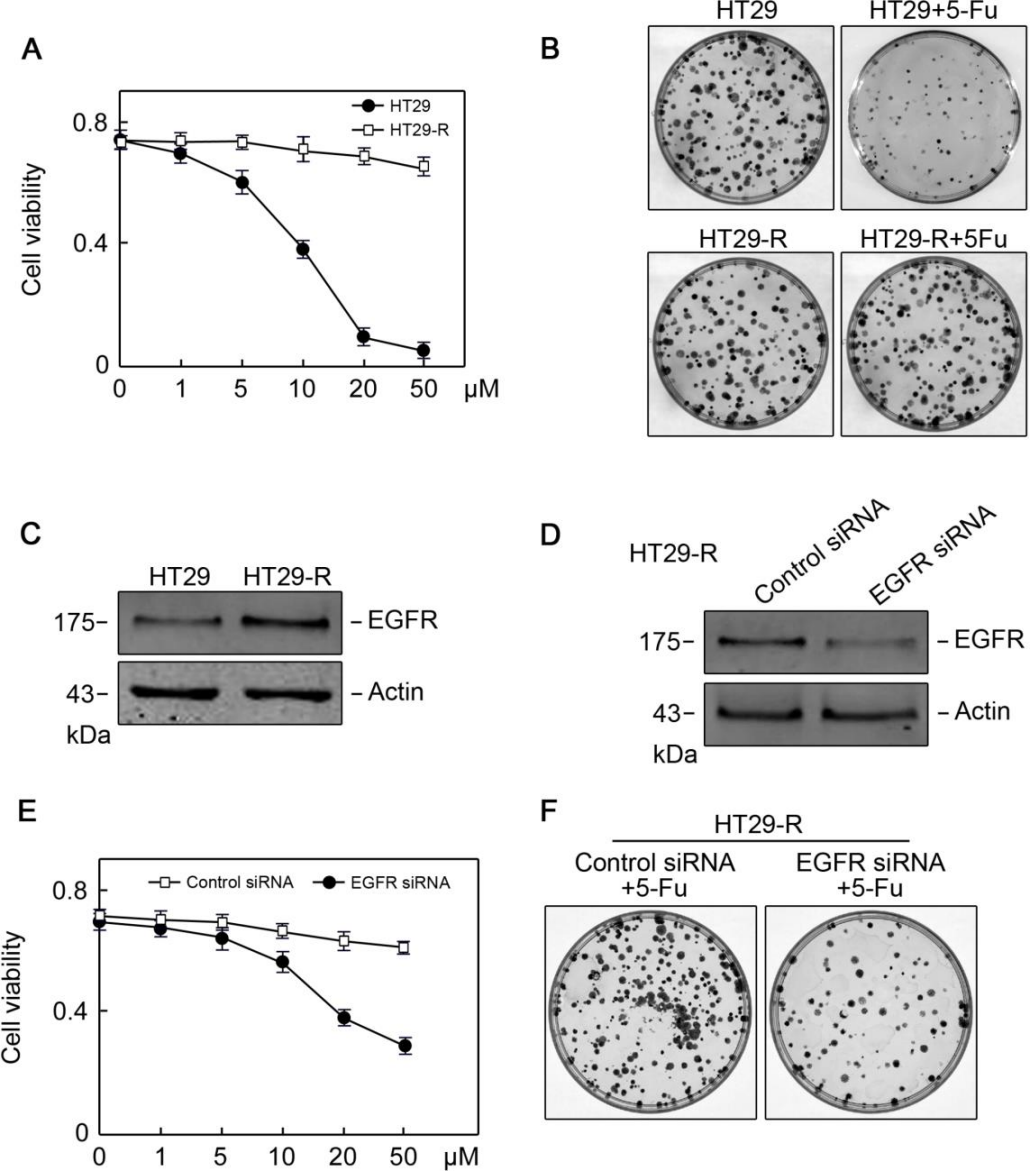
**EGFR contributes to 5-FU resistance in colon cancer cells**. (**A**) The 5-FU IC50 values were determined in HT29 and HT29R cells cultured in the presence of various concentrations of 5-FU (n=3). (**B**) Colony formation assays revealed that HT29-R was insensitive to 5-FU treatment (n=3). (**C**) EGFR protein levels were increased in HT29-R cells compared with parental HT29 cells. (D) EGFR siRNA successfully suppressed EGFR expression in HT29-R cells (n=3). (**E**) EGFR silencing sensitized HT29-R cells to 5-FU treatment (n=3. (**F**) Colony formation assays showed that HT29-R was sensitive to 5-FU treatment upon EGFR siRNA transfection (n=3).

**Figure 5:**
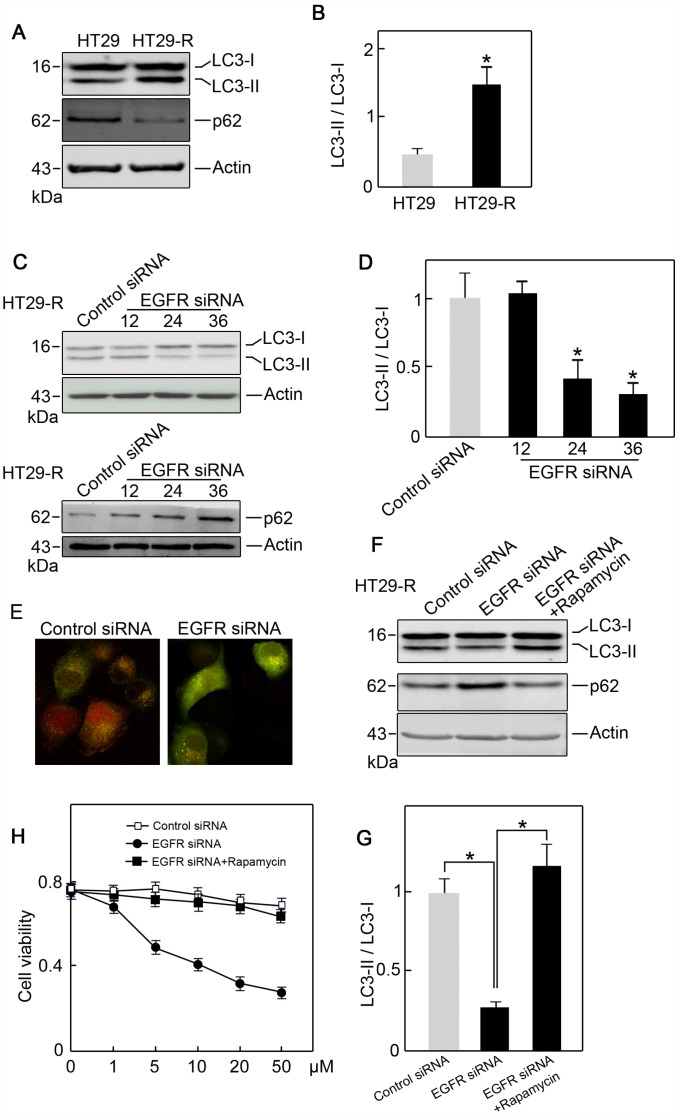
**EGFR contributes to 5-FU resistance in colon cancer cells through autophagy**. (**A**) Autophagy induction was increased in HT29-R cells compared with parental HT29 cells (n=3). (**B**) Conversion of LC3-I to LC3-II was increased in HT29-R cells compared with parental HT29 cells (n=3, *P < 0.05 versus control). (**C**) Knockdown of EGFR in HT29-R cells impaired autophagy flux (n=3). (**D**) Knockdown of EGFR in HT29-R cells inhibited the conversion of LC3-I to LC3-II (n=3, *P < 0.05 versus control). (**E**) Fluorescence images of mRFP-GFP-LC3 in HT29-R cells transfected with control siRNA or EGFR siRNA (200× magnification). (**F**) Rapamycin reversed autophagy inhibition induced by EGFR knockdown in HT29-R cells (n=3). (**G**) Rapamycin reversed the inhibition of LC3-I to LC3-II conversion caused by EGFR knockdown (n=3, *P < 0.05 versus control). (H) Rapamycin restored 5-FU resistance in HT29-R cells transfected with EGFR siRNA (n=3).

Combination treatment with anti-EGFR monoclonal antibodies and chemotherapy is a common strategy for the treatment of patients with colorectal cancer; however, the efficacy of this treatment is limited 10–20% of such patients [19]. To provide insights into combination treatment, we investigated the contribution of EGFR to 5-FU resistance in colon cancer. HT29 human colon cancer cells resistant to 5-FU were used as *in vitro* model. The IC50 of HT29 cells for 5-FU was 11.2 μM, whereas HT29-R cells were almost completely insensitive to 5-FU treatment (Figure 4A and 4B; Supplementary Figure 3A and 3B). HT29-R cells expressed EGFR at higher levels than parental HT29 cells (Figure 4C). Consistently, immunohistochemical staining revealed that the specimens from relapsed colon cancer exhibited higher levels of EGFR expression than adjacent tissues or untreated colon cancer tissues (Supplementary Figure 4A and 4B). In addition, the level of Beclin-1 was higher in relapsed colon cancer than that in untreated colon cancer tissues or adjacent tissues (Supplementary Figure 5A and 5B). We confirmed that transfection of EGFR-siRNA into HT29-R cells downregulated EGFR expression (Figure 4D). Knockdown of EGFR sensitized HT29-R cells to 5-FU treatment (Figure 4E; Figure 3C–D). Colony formation assays showed that HT29-R cell survival upon 5-FU treatment was dramatically lower in EGFR knockdown cells than that in control cells (Figure 4F). Next, we investigated whether EGFR regulated 5-FU resistance through autophagy. The results showed that p62 protein levels were lower and the LC3-II/LC3-I ratio was higher in HT29-R cells than that in HT29 cells, indicating that autophagy induction was increased in 5-FU resistant HT29-R cells (Figure 5A and 5B). EGFR silencing remarkably upregulated p62 and decreased the LC3-II/LC3-I ratio in HT29-R cells (Figure 5C and 5D). EGFR is closely related to autophagy, as determined by the correlation between EGFR and the autophagy marker LC3b. Autophagic flux could be monitored by the tandem-tagged LC3 construct mRFP-GFP-LC3, as the GFP fluorescence is lost while the mRFP fluorescence is more acid resistant. To examine whether EGFR knockdown impaired autophagic flux, EGFR siRNA or control siRNA transfected cells were infected by adenovirus carrying mRFP-GFP-LC3. After 24 h of infection, EGFR siRNA transfected cells tended to be yellow, whereas control siRNA transfected cells were predominantly red, reflecting less fusion of autophagosomes with lysosomes upon EGFR siRNA transfection compared with control siRNA (Figure 5E). To further confirm the role of the interaction between EGFR and autophagy in 5-FU resistance, HT29-R cells transfected with EGFR siRNA were treated with rapamycin, an autophagy inducer. We found that rapamycin could reverse the EGFR knockdown-induced autophagy inhibition (Figure 5F and 5G) and restored 5-FU resistance in HT29-R cells transfected with EGFR siRNA (Figure 5H).

### EGFR regulates 5-FU resistance in colon cancer cells in vivo

**Figure 6:**
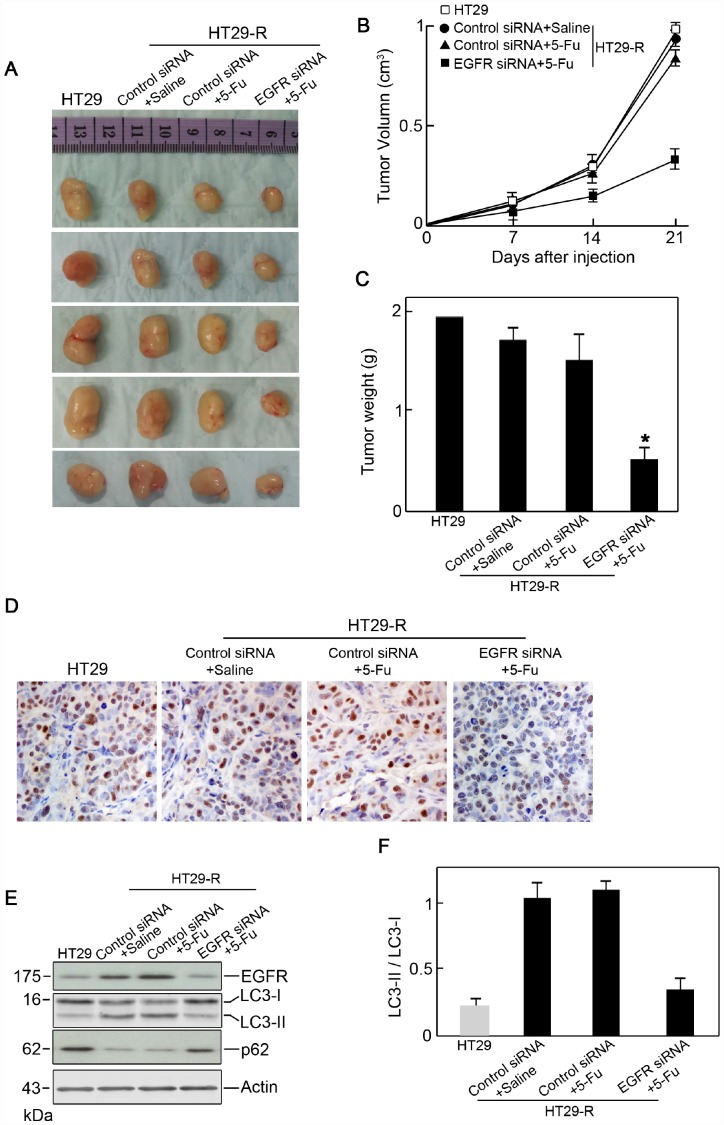
**EGFR contributes to 5-FU resistance in colon cancer cells through autophagy.** (**A**) Xenograft tumors formed in nude mice. A total of 5 × 10^6^ cells was subcutaneously injected into nude mice (n=5 for each group). The mice were sacrificed on day 21 after the injection. Tumors were harvested and representative images are shown. (**B**) and (**C**) EGFR knockdown promoted the anticancer effect of 5-FU *in vivo* as demonstrated by reduced tumor volume (**B**) and tumor weight (C) (n=5). (**D**) Immunohistochemical staining of Ki-67 in xenograft specimens. (**E**) Autophagy induction in a xenograft model was decreased by EGFR silencing upon 5-FU treatment. (n=5) (**F**) Conversion of LC3-I to LC3-II was inhibited by EGFR silencing upon 5-FU treatment (n=5, *P < 0.05 versus control).

To determine whether EGFR regulated 5-FU resistance *in vivo*, EGFR siRNA or control siRNA transfected HT29-R cells were subcutaneously injected into the flanks of nude mice. Mice were treated with saline or 5-FU by intraperitoneal injection. After 3 weeks, the mice were sacrificed and xenograft tumors were isolated. In mice injected with control siRNA transfected HT29-R cells, 5-FU treatment had a minor effect on reducing tumor size and weight (Figure 6A–6C). However, 5-FU treatment significantly reduced tumor size and weight in mice injected with EGFR-siRNA transfected HT29-R cells (Figure 6A–6C). Accordingly immunohistochemical staining in xenograft specimens revealed a much lower Ki-67 level in EGFR siRNA transfected HT29-R cells compared to other three groups (Figure 6D). In addition, the levels of EGFR, p62, and the LC3-II/LC3-I ratio were determined in xenograft tumors. The results showed that EGFR was downregulated by siRNA transfection (Figure 6E). Consistent with the *in vitro* results, xenografts from the EGFR-siRNA transfected group showed higher levels of p62 and a lower LC3-II/LC3-I ratio than those from the control siRNA transfected group (Figure 6E–6F).

### EGFR overexpression in 5-FU resistant colon cancer is regulated by RARA

**Figure 7:**
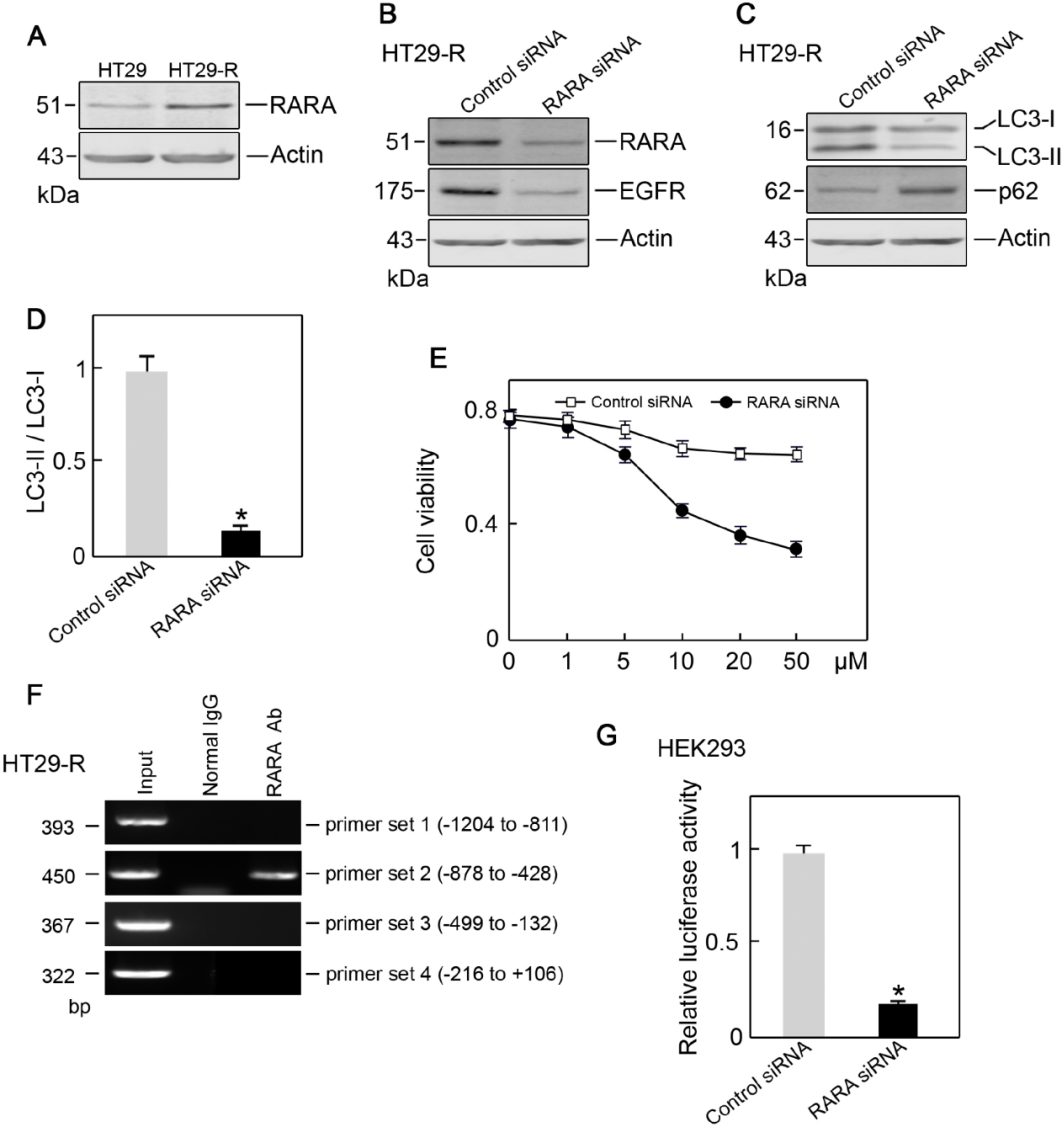
**RARA is the transcription factor for EGFR**. (**A**) RARA was upregulated in HT29-R cells compared with parental HT29 cells (n=3). (**B**) Knockdown of RARA decreased EGFR expression (n=3). (**C**) Knockdown of RARA blocked autophagy induction in HT29-R cells (n=3). (**D**) Knockdown of RARA inhibited the conversion of LC3-I to LC3-II in HT29-R cells (n=3, *P < 0.05 versus control). (**E**) Knockdown of RARA sensitized HT29-R cells to 5-FU treatment (n=3). (**F**) RARA binds to the EGFR promoter. ChIP assays were performed using anti-RARA antibody or IgG control. Representative data from three individual experiments are shown. (**G**) Knockdown of RARA reduced EGFR promoter activity as determined by the luciferase assay (n=3).

Since RARA was identified as a potential upstream regulator of EGFR, we next examined the level of RARA and its role in EGFR regulation. RARA protein expression was higher in HT29-R cells than that in HT29 cells (Figure 7A). The siRNA-mediated RARA knockdown downregulated EGFR expression (Figure 7B) and inhibited autophagy in HT29-R cells (Figure 7C and 7D). RARA knockdown sensitized HT29-R cells to 5-FU treatment with a lower IC50 (16.7 μM) (Figure 7E). ChIP and luciferase reporter assays were performed to determine whether RARA was a TF for EGFR. RARA cross-linked chromatin fragments prepared from HT29-R cells were immunoprecipitated using an anti-RARA antibody. ChIP enriched DNA samples were subjected to PCR using four sets of primers to analyze the fragments within the EGFR promoter region. The results showed one positive band at -878 to -428 in the EGFR promoter (Figure 7F). To investigate the effect of RARA on the regulation of EGFR promoter activity, the EGFR promoter region was cloned into the pGL-3 luciferase reporter vector. Analysis of luciferase reporter activity showed that siRNA mediated RARA downregulation significantly decreased EGFR promoter activity in HEK293 cells (Figure 7G).

## DISCUSSION

The correlation between alterations in cancer genomes and sensitivity to drugs has been reported [20]; however, the involvement of ATG genes remains unclear. In the present study, we used multi-dimensional omics data and clinical data in the NCI-60 cancer cell line to identify global alterations of ATG genes in different cancer types. The present results suggested that ATG genes may affect anticancer drug sensitivity in cancer cell lines according to large-scale pharmacogenomics data from CellMiner database. The present study provides a comprehensive analysis of the associations of ATG genes in different cancer types and highlights the potential clinical utility of ATG genes in cancer therapy. Autophagy is linked to the efficacy and toxicity of cancer treatments. For example, the autophagy marker LC3B is associated with poor overall survival in patients with hepatocellular carcinoma who are treated with sorafenib [21]. The combined use of autophagy inhibitors significantly increases the sensitivity of acute myeloid leukemia cells to cytarabine *in vitro* and *in vivo* [22]. We showed that ATG genes are highly correlated with the sensitivity to many anticancer drugs. All drugs tested were significantly correlated with the expression of at least one autophagy gene, and conversely, all ATG genes were significantly correlated with at least one drug. Melphalan was correlated with greatest number of ATG genes, including 38 positive correlations and 42 negative correlations. CAPN2 was correlated with the greatest number of drugs, including 3 positive correlations and 40 negative correlations.

Global interactions between ATG genes and CAGs were determined through co-expression analysis, PPI, and TF-target data, which led to the identification of a key sub-network with a |PCC| >0.3. The majority of CAGs were strongly correlated and interacted with ATG genes, suggesting that autophagy should be considered to target in cancer therapy. BRCA1 and ERBB2, which play key roles in breast cancer, were significantly correlated with 60 and 43 ATG genes, respectively. ErBB2 degradation by autophagy may alter the sensitivity to the humanized monoclonal antibody trastuzumab in breast cancer [23]. A high level of BRCA1 mutations in breast cancer suggests the activation of the autophagic and apoptotic machinery in response to fluorouracil, doxorubicin, and cyclophosphamide chemotherapy [24]. RARA and EGFR were the only TF-target pair identified in the sub-network. Several lines of evidence indicate that autophagy is regulated by EGFR in kinase-active and kinase-independent manners [25]. RARA triggers anti-proliferative effects in tumor cells by directly regulating gene expression [26]. The present results provided strong evidence that further efforts should be made to personalize or optimize cancer therapy by targeting ATG genes.

Autophagy could represent a new line of attack in the fight against cancer as reported previously [27]. Autophagy inhibition could counteract multidrug resistance by sensitizing cells to anticancer molecules [28]. For example, a combination therapy consisting of anti-EGFR monoclonal antibodies and autophagy inhibitors would represent a multi-pronged approach that could be developed into an active therapeutic strategy in metastatic colorectal cancer patients [29]. We showed that ATG genes have potential clinical relevance based on the associations between the expression levels of ATG genes and survival times. Several autophagy genes that may function as oncogenes, such as ERBB2, MEN1, and PDGFRB, and other ATG genes that may function as tumor suppressors, such as TP53, CEBPA, and PIK3R1 were identified. Many autophagy genes were associated with patient survival, suggesting that alterations of autophagy genes may occur during tumor progression. RARA and EGFR, which were identified as the only TF-target pair in the sub-network, were highly expressed in COAD and associated with worse survival in COAD patients. COAD is the third most common cancer worldwide, and metastatic disease accounts for 40–50% of newly diagnosed patients [30]. Colorectal cancers are resistant to treatment with anti-EGFR monoclonal antibodies such as cetuximab or panitumumab [31]. Our results highlight the possible clinical utility of targeting ATG genes in human cancer.

EGFR is a receptor tyrosine kinase involved in the pathogenesis and progression of many malignancies [32]. High EGFR gene copy number predicts poor outcomes in triple-negative breast cancer [33]. Overexpression of wild-type EGFR is tumorigenic and denotes a therapeutic target in non-small cell lung cancer [34]. EGFR alterations are also found in colon cancer [35]. EGFR regulates colon cancer stem-like cells during aging. Activation of EGFR results in unresponsiveness of colon cancer to BRAF(V600E) inhibition [36]. Feng et al*.* suggested that EGFR monoclonal antibodies could sensitize cancer stem cell-like colorectal carcinoma cells to 5-FU-induced apoptosis by affecting autophagy [37]. Consistent with previous studies, the present results showed that EGFR was overexpressed in 5-FU resistant HT29-R cells.

The results of the present study showed that 5-FU resistant HT29-R cells were characterized by increased autophagy induction. Silencing of EGFR reversed 5-FU resistance in HT29-R cells, whereas autophagy induction restored 5-FU resistance in HT29-R cells with knockdown of EGFR. These results suggested that EGFR regulated 5-FU resistance in HT29-R cells at least partially through autophagy induction. Recent studies demonstrated that EGFR signaling is involved in autophagy regulation [38]. EGFR-deregulated cells show increased dependence on autophagy for growth and survival [39]. EGFR tyrosine kinase inhibition induces autophagy in cancer cells [40]. Higher EGFR expression was indeed associated with lower autophagy induction. Active EGFR phosphorylates the autophagy protein Beclin 1 at multiple sites, leading to autophagy inhibition [41]. However, chemo-resistant cancer cells usually exhibit increased autophagy induction despite showing EGFR overexpression. Co-targeting EGFR and autophagy impairs cancer cell survival in response to chemotherapy.

Salvatori L et al*.* indicated that RARA could inhibit EGFR transactivation by competing with Sp1 for binding to the same promoter fragment in breast cancer cells [42]. However, in HT29-R cells, we found that RARA was positively correlated with EGFR expression. RARA binding to the EGFR promoter increased its activity. RARA knockdown reduced EGFR expression, sensitizing resistant HT29-R cells to 5-FU treatment. These results indicated that EGFR overexpression and its effect on 5-FU resistance in HT29-R were regulated by RARA overexpression.

In conclusion, the present study represents a comprehensive analysis of the global alterations of ATG genes in a broad range of cancer types, with emphasis on the roles of EGFR and RARA in colon cancer cells. The modulation of the autophagy process is a promising, but complex, therapeutic strategy to improve the efficacy of anticancer treatments. A better understanding of autophagy in tumor models is crucial for identifying new and effective therapeutic strategies for cancer treatment.

## MATERIALS AND METHODS

### Associations between drug activity and ATG genes

The ATG genes were obtained from three sources. The first source was the Human Autophagy database (HADb; http://www.autophagy.lu/), which provides a complete and up-to-date list of human genes and proteins involved directly or indirectly in autophagy as described in the literature. The second resource, Autophagy database (http://autophagy.info/), provides up-to-date information on basic ATG genes and their homologs in 41 eukaryotes in the relevant literature. The third resource was the Autophagy Regulatory Network (ARN; http://arn.elte.hu/), which contains manually curated, imported, and predicted interactions of autophagy components in humans. Using a combination of the three resources, we identified 770 ATG genes. Data on drug activity and the expressions of ATG genes were obtained from the NCI-60 cancer cell line database, which is a large-scale information dataset with multiple genomic and drug response platforms. Potential associations between drug activity and the expression levels of ATG genes were retrieved using CellMiner [43], which is a powerful platform that allows rapid data retrieval of gene transcripts along with activity reports for chemical compounds. CellMiner provides ‘NCI-60 Analysis Tools’ to allow rapid data retrieval of transcripts for 22,379 genes and 360 microRNAs along with activity reports for 20,503 chemical compounds [43]. Thus, we could obtain the relationships between mRNA expression and the 50% growth inhibitory concentration values of drugs by calculating the Pearson correlation coefficient (PCC) between them. U.S. food and drug administration (FDA)-approved drugs and clinical trial drugs were selected, and drugs that were not listed in the DrugBank database were filtered out [44]. To retrieve the correlations, we took the following steps: (i) in the ‘NCI-60 Analysis Tools’ page, click ‘Pattern comparison’ and ‘Drug NSC#’ option in Step 1 section; (ii) input the drug NSC ID in the Step 2 section; (iii) enter e-mail address and CellMiner sends the result documents of Pearson correlations between all genes and each input drug; (iv) integrate all drug files together and (v) filter the relationships between drug and ATG genes by PCC |R| > 0.3 as well as ATG genes significantly correlated with at least 10 drugs.

### Interactions and correlations between ATG genes and clinically actionable genes

To obtain a list of CAGs, we first obtained 132 genes from a previous study (http://software.broadinstitute.org/cancer/cga/target). To examine the correlation between transcriptional expression of ATG genes and CAGs in tumor samples, Pearson’s correlation coefficients were calculated based on the expression levels of 60 cancer cell lines in CellMiner. To obtain the sub-network, |R| > 0.3 and *p* < 0.01 were considered as the threshold. Protein-protein interaction (PPI) data were downloaded from the Human Protein Reference Database (HPRD; http://www.hprd.org/). The relationships between TFs and target genes were obtained from TRANSFAC [45].

### Identification of clinically relevant ATG genes

Clinical information for cancer patients, including overall survival, disease stage, and tumor subtypes, were obtained from the TCGA data portal (http://gdac.broadinstitute.org/). Multivariate analyses were performed to identify significant independent ATG genes for prognosis prediction. The hazard ratio (HR) for each factor was calculated using the Cox regression proportional hazards model, and median OS was calculated using a Kaplan-Meier survival analysis. A multivariate analysis was performed using factors with *p*-values <0.05, as identified in the univariate analysis. A log-rank test was used to compare the survival curves, and *p* < 0.05 was considered statistically significant. The GEPIA database (http://gepia.cancer-pku.cn/) was used to investigate the correlation between the expression of each gene in the sub-network and tumor stage.

### Cell culture

HT29 colon cancer cells were obtained from the Cell Bank of the Chinese Academy of Sciences (Shanghai, China), and cultured in Dulbecco’s modified Eagle medium (Invitrogen, Carlsbad, CA, USA), supplemented with 10% fetal bovine serum (FBS; Invitrogen), 50 U/mL penicillin, and 50 μg/mL streptomycin (Invitrogen). Cells were maintained at 37°C in a humidified incubator at 5% CO_2_.

### Generation of 5-FU resistant HT29 cells

5-FU-resistant cell lines were generated from HT-29 cells. Briefly, parental cells were treated with gradually increasing concentrations of 5-FU under regular cell culture conditions for the selection of resistant cells. After successive treatments for up to 3 months, resistant cell clones were pooled and used for all subsequent experiments. To ensure maintenance of resistance to 5-FU, HT29R cells were steadily grown in the presence of 10 μM 5-FU.

### Acridine orange/ethidium bromide (AO/EB) fluorescence staining

HT29 or HT29-R cells were seeded in 6-well plates and treated with the indicated reagents. The cells were incubated with acridine orange and ethidium bromide mixing solution for 5 min (Solarbio Biotechnology, Beijing, China). Cellular morphological changes were examined by fluorescence microscopy at 200× magnification. The percentage of apoptotic cells was calculated using the following formula: apoptotic rate (%) = number of apoptotic cells/ total number of cells counted.

### Clinical tissues and immunohistochemistry

Colon cancer tissues and adjacent tissues were collected from surgical resections in the Third Affiliated Hospital of Harbin Medical University. Informed consent was obtained from all patients, and the research method was approved by the Ethics Committee of Harbin Medical University. Tissue samples were immediately fixed in 4% paraformaldehyde for 24 h. Tissues were permeabilized with PBS-T for 20 min and incubated in 0.3% hydrogen peroxide for 20 min to quench endogenous peroxidases. After washing, tissues were blocked with 5% normal goat serum (Invitrogen, USA) for 30 min and incubated overnight at 4°C with anti-EGFR antibody (Cell Signaling Technology, USA). The following day, specimens were washed and incubated with a biotin-binding secondary antibody for 20 min. After washing, the specimens were developed with 0.05% DAB (Sigma-Aldrich, Oakville, ON, Canada) and 0.03% H_2_O_2_, counterstained with hematoxylin, dehydrated in increasing ethanol concentrations, cleared with xylene, and then coverslipped.

Stained specimens were examined using an Olympus BX51microscope. Digital images were analyzed using Image-Pro Plus 6.0 software.

### siRNA transfection

The sequences of the EGFR siRNA (Cat# B02003) and RARA siRNA (Cat# B03001) and its corresponding negative controls were synthesized by GenePharma (Shanghai, China).

HT29-R cells were seeded into 60 mm plates 24 h prior to transfection. EGFR siRNA or RARA siRNA or their corresponding negative control were transfected using Lipofectamine 2000 (Invitrogen, USA) with serum-free medium according to the manufacturer’s protocol. At 5 h after transfection, cells were changed to complete medium and subsequently cultured for 48 h. The cell lysates were harvested 48 h after transfection.

### Protein isolation and western blotting

Cells were lysed with RIPA lysis buffer containing a protease inhibitor cocktail (Roche, Switzerland). Equal amounts of protein were separated by SDS-PAGE and transferred to PVDF transfer membranes (Thermo Scientific, USA). After blocking, the blots were probed with primary antibodies to EGFR, LC3, Beclin-1 (Cell Signaling Technology, USA), RARA (Abcam), and Actin (Santa Cruz, USA). After washing and incubating with rabbit or mouse secondary antibodies (Cell Signaling, Technology, USA), the blots were visualized using the ECL reagent (GE healthcare, USA).

### CCK-8 cell viability assay

HT29 or HT29-R cells treated with the indicated reagents were seeded into 96-well plates at a density of 5 × 10^3^ cells per well and cultured for 48 h. Cell viability was assessed using the Cell Counting Kit-8 (CCK-8, Dojindo, Japan).

### Colony formation assay

HT29 or HT29-R cells (8 × 10^2^) treated with the indicated reagents were seeded into 6 cm dishes. After 10 days of culture, colonies were stained with 0.1% crystal violet in 20% methanol for 15 min. The samples were photographed, and the numbers of visible colonies were counted.

### Autophagic flux assessment by tandem mRFP-GFP-LC3

Adenovirus carrying mRFP-GFP-LC3 was purchased from Hanbio Co. LTD. (Shanghai, China). To examine autophagic flux, HT29-R cells were transfected with control siRNA or EGFR siRNA. After 24 h of transfection, mRFP-GFP-LC3 adenovirus was applied to the cells and the medium was changed after 12 h. Fluorescence images were captured at 24 h after infection. The GFP signal is sensitive to the acidic conditions of the lysosome lumen, whereas mRFP is more stable. Therefore, colocalization of both GFP and mRFP fluorescence indicates a compartment that has not fused with a lysosome. By contrast, an mRFP signal without GFP indicates a compartment fused with a lysosome.

### Animal experiments

Athymic BALB/c nude mice (6 weeks old) were obtained from Vital River Laboratory (Beijing, China). Mice were maintained under specific pathogen-free conditions, housed in isolated vented cages, and handled using aseptic procedures. Mice were injected subcutaneously in the right shank with 5 × 10^6^ of 5-FU resistant HT-29-R cells transfected with control siRNA or EGFR siRNA. All mice were treated with saline or 5-FU by intraperitoneal injection every 2 days. Tumor size was measured using a caliper every 3 days. Both the maximum (L) and minimum (W) lengths of the tumors were measured, and the tumor size was calculated as ½LW2. After 3 weeks, mice were sacrificed, and the xenografts were isolated, weighed, and used for further experiments.

### Chromatin immunoprecipitation (ChIP) assay

HT29-R cells were trypsinized and harvested in a centrifuge tube. Cells were washed in PBS, fixed with 0.5% formaldehyde at room temperature for 10 min and quenched by addition of 125 mM glycine. Fixed cells were suspended in sonication buffer (20 mM Tris-HCl pH 8.0, 1 mM EDTA, 1 mM DTT, and 0.02% SDS), and sonicated with Bioruptor (Scientz, China) to fragment chromatin DNA. The soluble chromatin was recovered by centrifugation, diluted with two volumes of IP buffer (30 mM Tris-HCl pH8.0, 450 mM NaCl, 0.75 mM EDTA, 0.75 mM DTT, 1.5% Triton X-100, 0.075% SDS, and 7.5% glycerol) as input samples. Samples were immunoprecipitated with RARA antibody (Abcam) pre-incubated with Protein A/G PLUS-Agarose (Santa Cruz, USA) and then washed twice with PBS. IP enriched DNA was incubated at 65°C to de-crosslink. The purified DNA was analyzed by real-time PCR (Applied Biosystems, USA) with Power SYBR Green PCR master Mix (Life Technologies, USA).

Primers for ChIP were as follows: Primer1-F: CTCCCCTTCAGAGACAGCAAAG; Primer1-R: CTTCGCAAAAGTGAAGCTCTTG; Primer2-F: CCTCTCTAAAAGCACCTCCACG; Primer2-R: TTCCCCCTTTCCCTTCTTTTG; Primer3-F: TCTAAGGCTCGGCCAGTCTGTC; Primer3-R: ACCAGGCGGCGGAGGAGGGATC; Primer4-F: TTGGGTCCCCGCTGCTGGTTC; Primer4-R: GGTTGTGGCGTTGGCGGCGAGG.

### Luciferase reporter assay

Luciferase activity was analyzed using luciferase reporter assays. The EGFR promoter sequence was cloned into the pGL-3-basic firefly luciferase reporter and co-transfected into HEK293 cells with the pTK-Renilla luciferase construct (for normalization). Cell extracts were harvested at 48 h after transfection, and luciferase activity was measured using the Dual-Luciferase Reporter Assay System (Promega, USA) as described in the manufacturer’s protocol.

### Data analysis

Data were obtained from at least three independent experiments and are presented as the mean ± standard deviation. Statistical data were analyzed using Statistical Program for Social Sciences (SPSS) 17.0 software (SPSS, Chicago, IL, USA). Data were evaluated by unpaired Student’s t test. *p* < 0.05 was considered to represent a significant difference.

### Statistical analysis

A *p*-value of 0.05 was used as the cut-off value for statistical significance. Software “R” version 3.2.3 was used for the statistical analysis.

## Supplementary Material

Supplementary Figures
