# Supplementary material for: Over-expression of EGFR regulated by RARA contributes to 5-FU resistance in colon cancer
Source: Aging (Albany NY). 2020 Jan 2;12(1):156–77. doi: 10.18632/aging.102607 (PMC6977699; doi:10.18632/aging.102607)
Supplement: Supplementary Figures [file aging-12-102607-s001..pdf]

SUPPLEMENTARY FIGURES

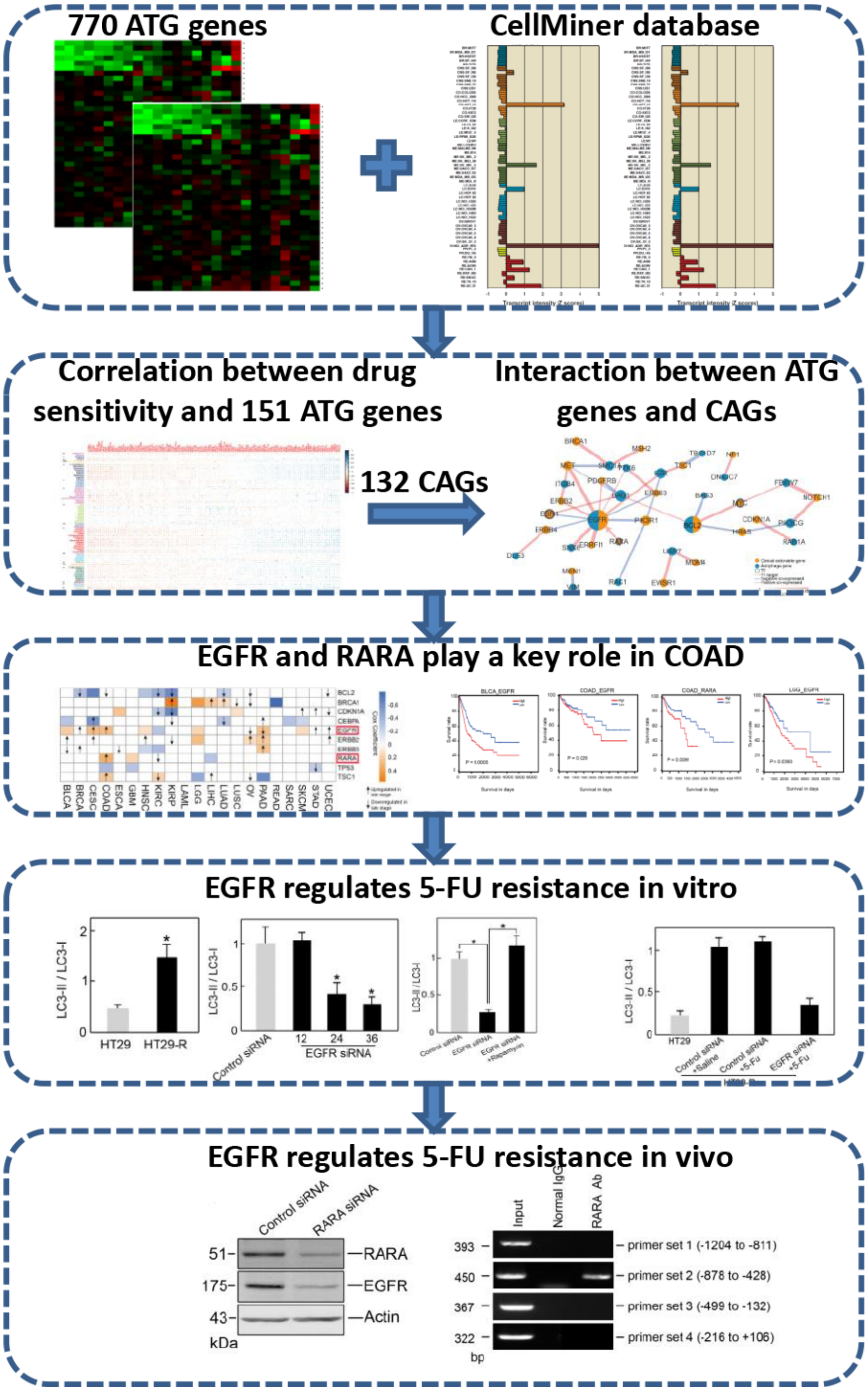

Supplementary Figure 1. The sketch map of this study.

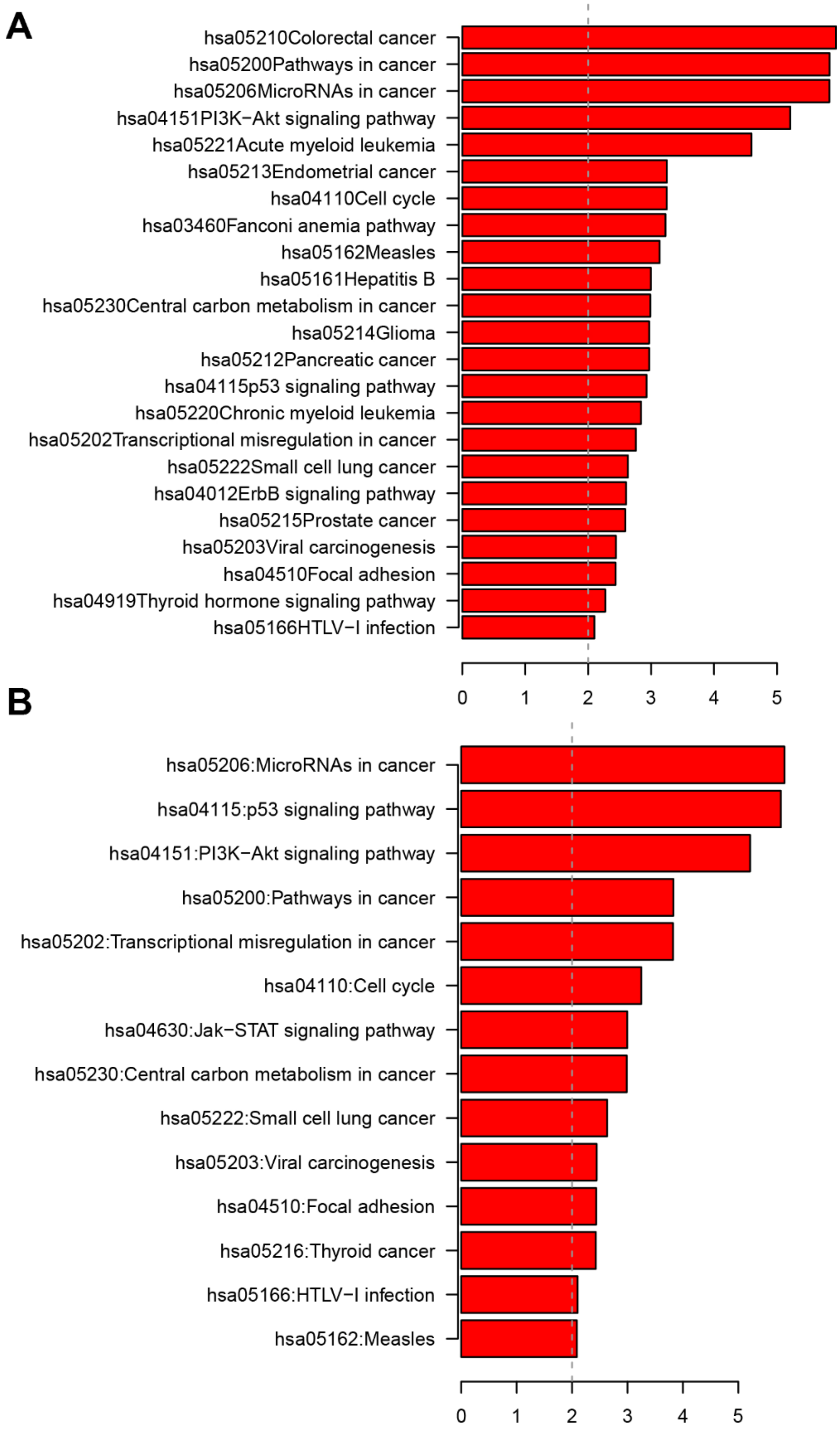

Supplementary Figure 2. Pathway enrichment analysis of significantly correlated genes of (**A**) CDC42BPB and (**B**) KIF21B.

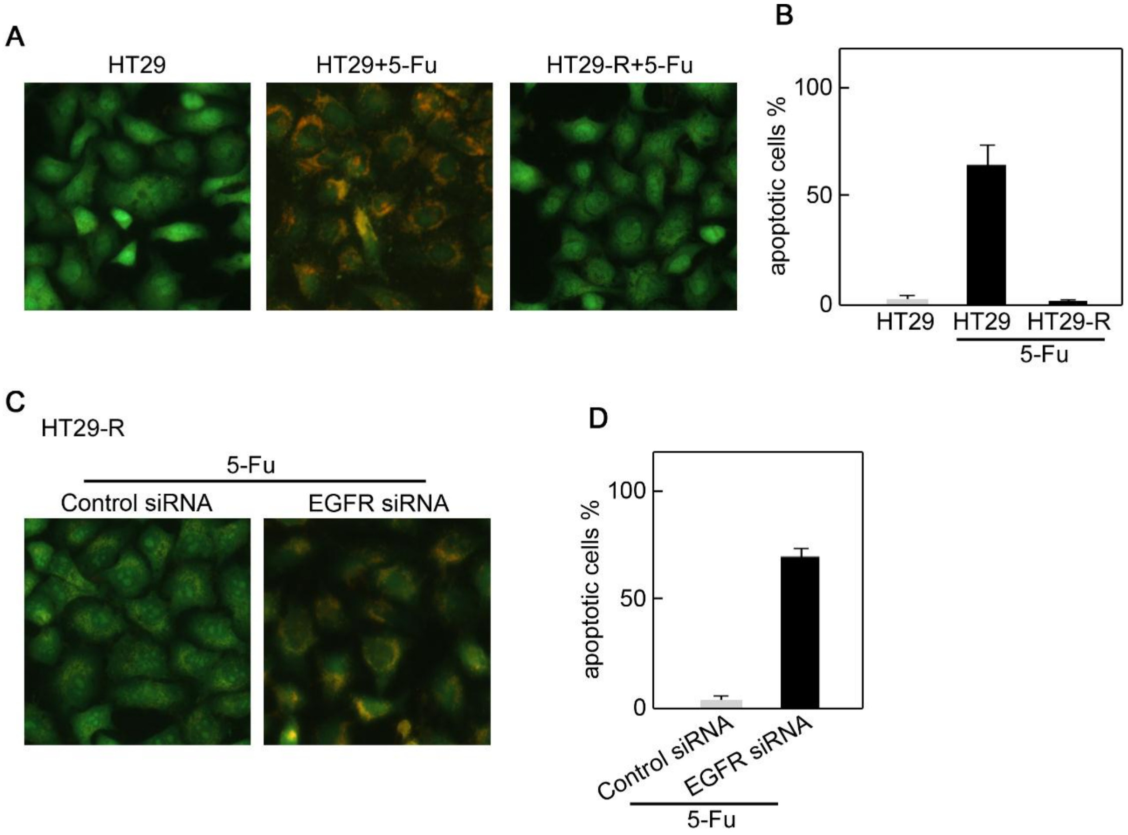

Supplementary Figure 3. Silencing of EGFR increased the anticancer effect of 5-FU in HT29-R cells. (**A**) AO/EB staining showed that HT29-R cells were insensitive to 5-FU treatment. (**B**) The proportion of apoptotic cells analyzed from A. (**C**) Silencing of EGFR increased the anticancer effect of 5-FU in HT29-R cells. (**D**) The proportion of apoptotic cells analyzed from (**C**).

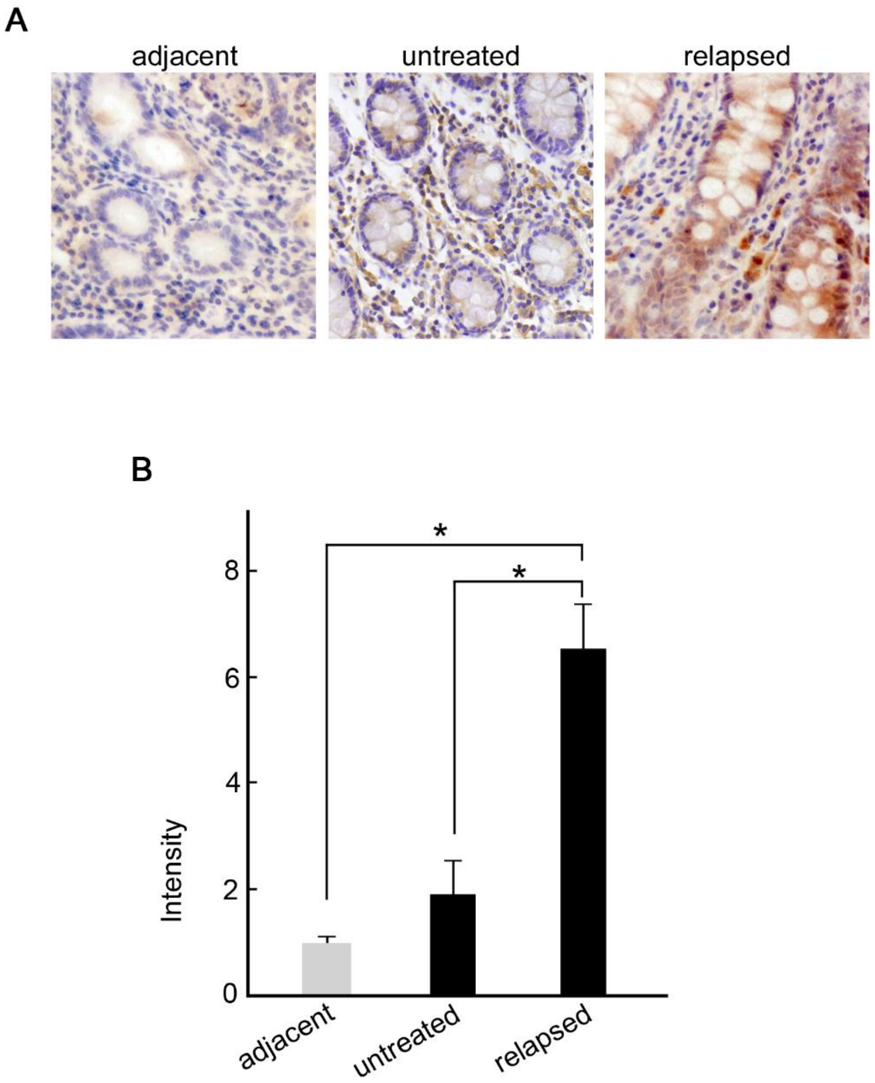

Supplementary Figure 4. EGFR was overexpressed in relapsed colon cancer tissues. (**A**) EGFR expression was determined by immunohistochemical staining. Adjacent: adjacent tissue; untreated: primary colon cancer without any treatment; relapsed: relapsed colon cancer after 5-FU treatment. (**B**) The positive staining intensity was assessed from A.

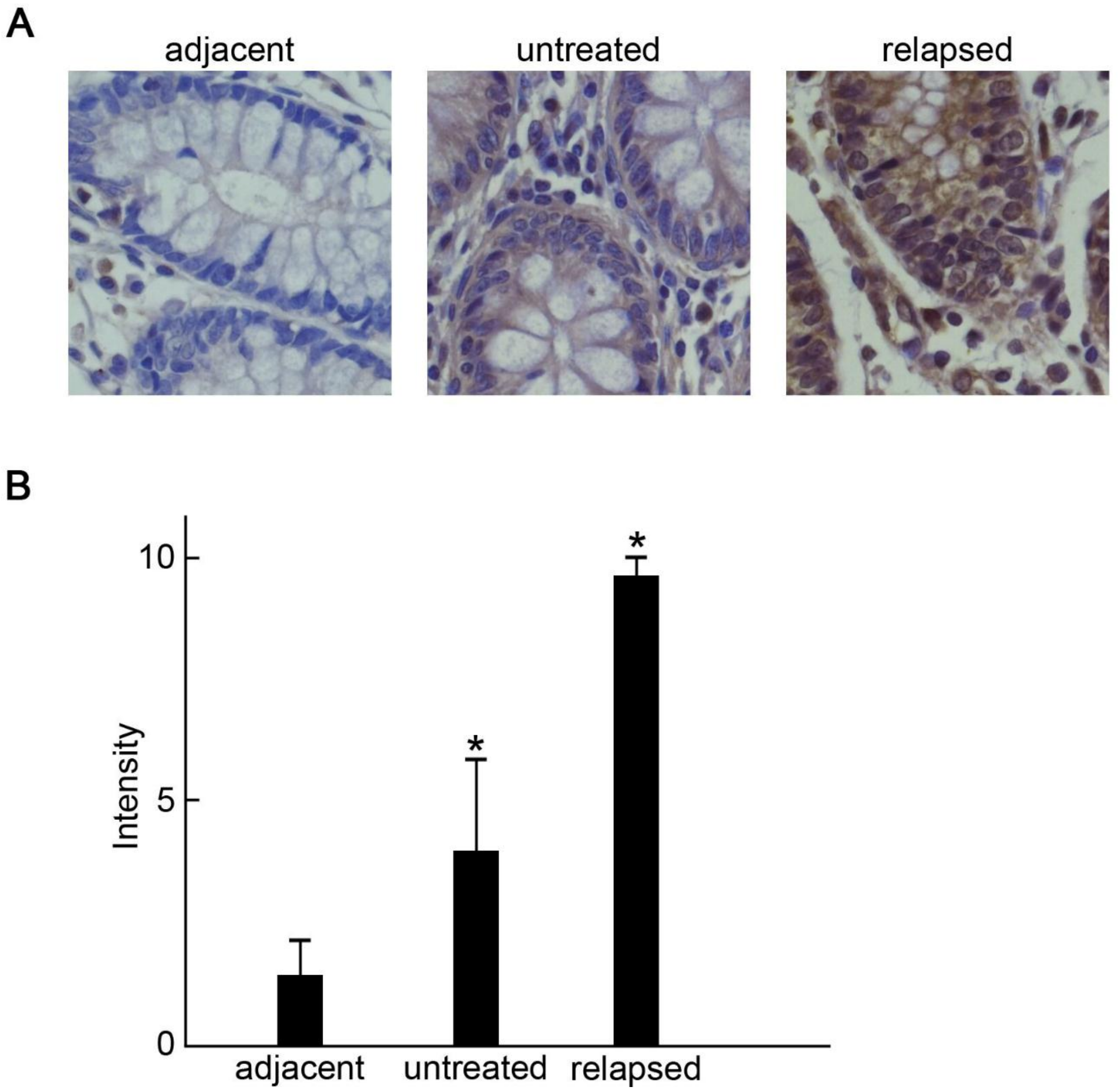

Supplementary Figure 5. p62 was decreased in relapsed colon cancer tissues. (**A**) p62 expression was determined by immunohistochemical staining. Adjacent: adjacent tissue; untreated: primary colon cancer without any treatment; relapsed: relapsed colon cancer after 5-FU treatment. (**B**) The positive staining intensity was assessed from (**A**).
